# The Shock Effect of Inorganic Suspended Solids in Surface Runoff on Wastewater Treatment Plant Performance

**DOI:** 10.3390/ijerph16030453

**Published:** 2019-02-04

**Authors:** Li He, Tao Tan, Zhixi Gao, Leilei Fan

**Affiliations:** 1 College of Resources and Environment, Zunyi Normal University, Zunyi 563006, China; wyhgzx@163.com (Z.G.), fldxx@126.com (L.F.); 2 Chongqing Architectural Design Institute of China, Chongqing 400015, China; tantao13594037122@163.com

**Keywords:** surface runoff, inorganic solids, wastewater treatment, MLVSS/MLSS

## Abstract

Previous studies on the water quality of surface runoff often focused on the chemical oxygen demand (COD), nitrogen, phosphorus, and total suspended solid (TSS), but little is known in terms of the inorganic suspended solids (ISS). This research investigated the effects of ISS carried by surface runoff on the treatment efficiency of the pretreatment facilities and the ratio of mixed liquor volatile suspended solid to mixed liquor suspended solid (MLVSS/MLSS) of the activated sludge in a wastewater treatment plant (WWTP) with the anaerobic-anoxic-oxic (AAO) process in Chongqing city, China. The results showed that the surface runoff had a long-lasting impact on the grit removal capacity of the grit chamber, affecting the normal operation after the rainfall. In contrast, the primary sedimentation tank showed strong impact resistance with higher removal rates of COD, TSS, and ISS. Nonetheless, the primary settling tank aggravates the removal of organic carbon in sewage during rainfall, having a negative impact on subsequent biological treatment. The ISS in the surface runoff could increase the sludge concentration and decrease the MLVSS/MLSS ratio. After repeated surface runoff impact, the MLVSS/MLSS ratio in the activated sludge would drop below even 0.3, interrupting the normal operation of WWTP.

## 1. Introduction

In general, urban surface runoff contains toxic and harmful pollutants (such as suspended solids, vehicle emissions, air sedimentation, etc.), which might be significantly greater than those of urban sewage. Such circumstances are receiving widespread attention [1,2,3,4]. A survey of water pollution in the United States in 1990 reported that over 30% of the water bodies were contaminated by non-point source pollution [5]. Studies have found that somewhere between 50% and 60% of the suspended solids in the combined drainage outflows originate from urban surface runoff [6,7]. In some river basins, the concentration of suspended solid could be 22–106-times above the typical values [6].

Recently, a number of studies have focused on investigating the impacts of urbanization degree [8,9], seasonal variation [10,11], and land use [12,13] on surface runoff pollutants and the contribution of surface runoff to pollutants in the combined drainage system [7,14]. In these studies, researchers paid the most attention to the nutrient contaminants (such as chemical oxygen demand (COD), total nitrogen (TN), total phosphorus (TP)) [15,16], total suspended solids (TSS) [17], and heavy metals [18] in surface runoff. To our knowledge, few studies have evaluated the contents of inorganic suspended solids (ISS) in surface runoff. Several studies suggested that the ISS/TSS ratios during rainy days could reach as high as 60% in the combined drainage system [19]; about 10% of the fine sand in bed sediments came from surface runoff [20]. These indicate that the content of inorganic solids is quite substantial in the runoff. 

With the widespread application of the sequencing batch reactor (SBR) and oxidation ditch technology in wastewater treatment processes, many wastewater treatment plants (WWTP) have stopped using the primary settling tank [21]. However, most of the cities in China continue to apply the combined drainage system. In this case, inorganic solids in surface runoff that cannot be removed by the grit chambers would enter the biochemical treatment tanks directly, causing the deposition of ISS and reduction in the mixed liquor volatile suspended solid to mixed liquor suspended solid (MLVSS/MLSS) ratio of activated sludge. In a sense, high contents of ISSs in surface runoff could significantly reduce the performance of WWTP. However, such a kind of investigation has not been performed elsewhere.

In this paper, a wastewater treatment plant (WWTP) in Chongqing city (China) is selected as a test bed to perform a pilot scale study. The aim is to understand how a “shock load” of inorganic solids in surface runoff would affect the water quality and sludge activity ratio. This study should provide some useful scientific basis for refining and improving the regulation and operation of WWTP.

## 2. Materials and Methods 

### 2.1. Introduction of WWTP

**Figure 1:**
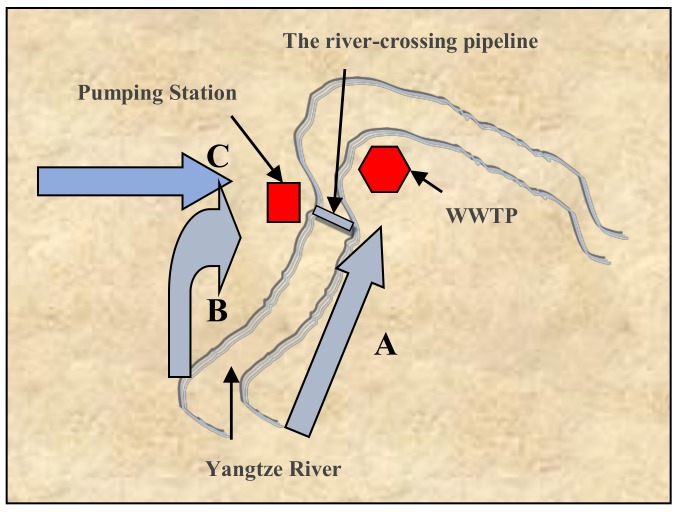
The wastewater collection route. WWTP, wastewater treatment plant.

**Figure 2:**
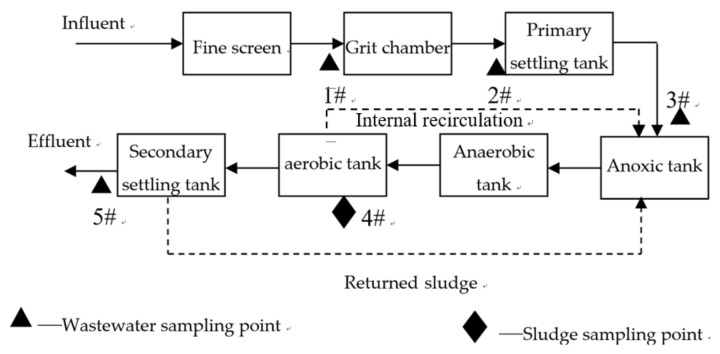
Schematic diagram of WWTP and sampling locations.

The selected WWTP is located in Chongqing city (China) with a combined sewage collection system. The WWTP provides wastewater treatment services for three nearby districts, including Nan’an District, Jiulongpo District, and Yuzhong District (see Figure 1). The wastewater from Nan’an District enters the WWTP directly through Route A; and the wastewater from Jiulongpo District and Yuzhong District is channeled to the pump station through the Route B and Route C, respectively. During rainy days, the pumping station regulates the flow by controlling the number of sewage pumps. The wastewater collection route is shown in Figure 1. The treatment capacity of the WWTP in dry and rain seasons is 60,000 m^3^/d and 100,000 m^3^/d, respectively. The inverted anaerobic-anoxic-oxic (AAO) process is used, and the flow chart is shown in Figure 2.

### 2.2. Rainfall

**Figure 3:**
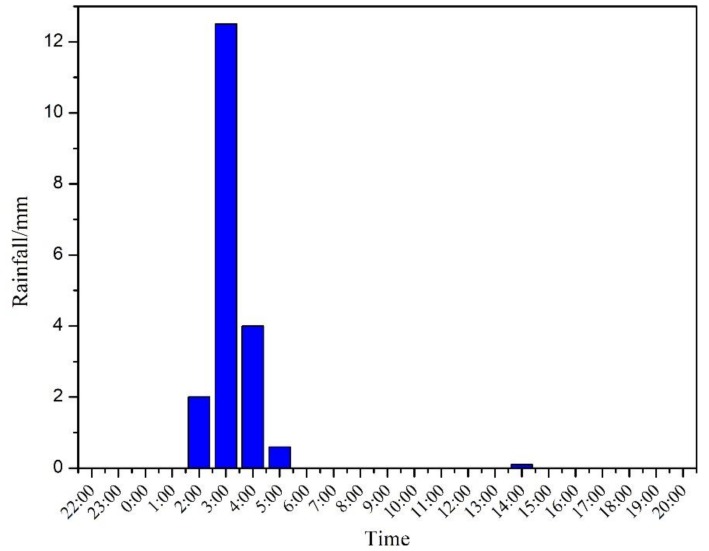
Rainfall variation.

On 29 May, the rainfall mainly occurred between 2:00 and 4:00; while the hourly rainfall hit a peak of 12.5 mm at 3:00 (see Figure 3). According to the U.S. Geological Survey (USGS) definition of individual rainfall events, this rainfall can be categorized as heavy rain, and the surface erosion rate was greater than or equal to 90% [18]. Prior to this rainfall event, it had been dry and sunny over the past 15 days. Therefore, the surface runoff generated by this heavy rainfall event should be representative in the current study.

### 2.3. Sampling Method

The locations of four water quality sampling points and one sludge sampling point are shown in Figure 2. An automated water sampler (YCS-778, Suzhou Yuchen Instrument Co. LTD., Suzhou, China) was used to take mixed samples.

The sampling process was divided into three stages. The first stage was the background period, which was five days before the rain. The second stage was the impact period, which was from the beginning of the rainfall to the end of the shock impact of the system, lasting from 0–400 min. The third stage was the recovery period, which started right after the end of the rainfall and until the MLVSS/MLSS ratio of the mixed liquor recovered to the background value, lasting from 440 min to the 8th day.

#### 2.3.1. Sampling Plan during Impact Period

The purpose of setting up 1#~3# sampling points was to investigate the treatment efficiency of the pretreatment facilities of WWTP. Samples were taken every 20 min between 0 h and 4 h since the beginning of the rainfall event; then, every 40 min between 4 h and 8 h; every one hour between 8 h and 12 h; and finally, every 2 h between 12 h and 20 h.

Taking into account the hydraulic retention time of sewage in the biological treatment structures, the sampling intervals of 4# and 5# should be increased accordingly. Samples were taken every one hour between 0 h and 8 h since the beginning of the rainfall event; then every two h between 8 h and 16 h; and at last, every 4 h between 16 h and 20 h.

#### 2.3.2. Sampling Plan during the Background and Recovery Periods

The sampling started at 8:00 and was taken every 4 h. The six samples collected over a day were mixed evenly for index test.

### 2.4. Test Indicators and Methods

TP and TN were tested in accordance with the national standard methods (State Environment Protection Administration of China, 2002). The COD was determined using a DR1010 COD Analyzer (HACH, Loveland, CO, USA). Particle size was determined using a laser particle size analyzer (BT-9300HT, Bettersize Instruments LTD., Dandong, China). All samples were passed through a 0.45-µm filter and dried to a constant weight at 105 °C to obtain MLSS. Inorganic solids (MLISS) were also determined by weighing following incineration at 600 °C for 2 h. Organic solids (MLVSS) were calculated as the difference between MLSS and MLISS.

## 3. Results

### 3.1. Variation of Pollutants Concentration in Different Periods

**Figure 4:**
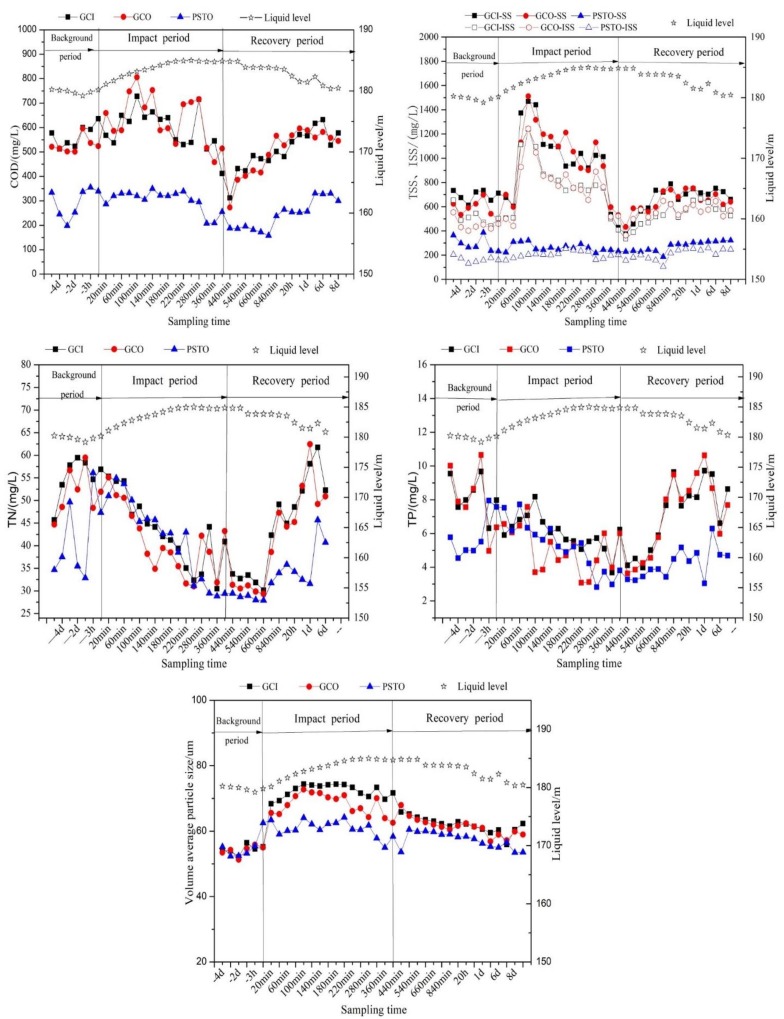
The variation of pollutant concentration in different periods. ISS, inorganic suspended solids; AAO, anaerobic-anoxic-oxic. Grit Chamber Influent Grit Chamber Influent—GCI; Grit Chamber Effluent—GCO; Primary Settling Tank Effluent—PSTO. Meanwhile, PSTO is also the AAO process influent.

Figure 4 shows the variation of pollutant concentration in different periods. The specific values are shown in Table 1. The liquid level (This refers to the water level of the sewage pump station before the coarse screen. These are the on-line monitoring data, which can reflect the flow change after the runoff entering the WWTP.) of the WWTP increased gradually with the rainfall and then maintained at the highest level during 200–480 min (which is somewhere between 184.85 m and 185.00 m). At 80 min since the rainfall started, the concentrations of COD, TSS, and ISS elevated to the highest levels, which were 728 mg/L, 1469 mg/L, and 1242 mg/L, respectively. Relative to the mean values of the background period, the COD, TSS, and ISS concentrations increased by 30.7%, 113.5%, and 139.8%, respectively. Because of the scouring effect of the initial rainwater, the peak value of pollutant concentration was ahead of the peak value of runoff, and the pollutant load mainly concentrated at the rising stage of runoff. This is consistent with previous studies. Subsequently, it decayed exponentially between 100 min and 440 min and gradually stabilized at the concentration of the background period. After the rain water entered WWTP, the concentrations of TN and TP kept falling. When the flow returned to the normal level, the concentrations of TN and TP gradually recovered.

### 3.2. Pollutant Removal Efficiency of the Pretreatment Facilities of WWTP

**Figure 5:**
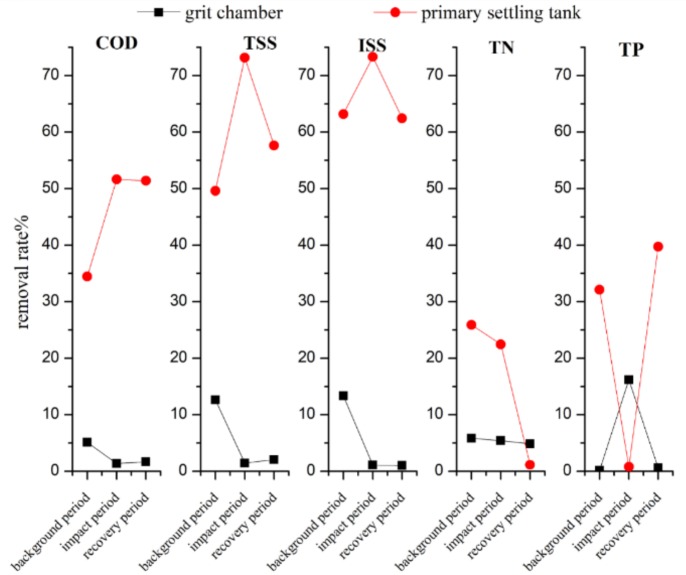
The removal rate of pretreatment facilities.

The removal rates of pretreatment facilities in different periods are shown in Figure 5. In the background period, the removal rates of COD, TSS, and ISS in the grit chamber were 5.21%, 12.65%, and 13.33%, respectively. In the impact period, they decreased to 0.00%, 0.00%, and 1.18%, respectively. Nonetheless, the pollutant removal capacity of the grit chamber was not recovered during the recovery period. This suggests that surface runoff has a significant impact on the grit-removal capacity of the grit chamber. This impact is persistent with a lasting effect on the normal operation of the grit chamber after the rainfall event. 

Surprisingly, the primary sedimentation tank demonstrated very good impact resistance during the impact period. In this period, the removal rates of COD, TSS, and ISS in the primary settling tank escalated from 45.64%, 49.59%, and 63.25% to 51.52%, 73.12%, and 73.27%, respectively. According to the data in Table 1, it can be found that the removed concentration of TSS by the primary settling tank was 298, 710, and 372 mg/L during the background period, impact period, and recovery period, and the ISS removal concentration was 284, 551, and 348 mg/L, respectively. These results show that the primary settling tank ordinarily has the same performance on TSS and ISS removal, but it does remove 149mg/L of VSS during the rainfall event. Combined with the increase of the COD removal rate, it can be inferred that the primary settling tank aggravates the removal of organic carbon in sewage during rainfall, which has a negative impact on subsequent biological treatment.

The TSS/COD, ISS/COD, COD/TN, and COD/TP ratios of the grit chamber influent, effluent, and primary settling tank effluent (or AAO influent) are shown in Table 2, Table 3, and Table 4, respectively. The TSS/COD ratio of the influent, the effluent of the grit chamber, and the effluent of the primary sedimentation tank in the background period were 1.24, 1.14, and 1.06, respectively; and their mean ISS/COD ratios were 0.93, 0.85, and 0.57, respectively. After the rainfall, the TSS/COD and ISS/COD ratios of the influents of the grit chamber, primary sedimentation tank, and AAO peaked at 80 min. Their maximum values of TSS/COD ratios were 2.35, 2.02, and 1.18, respectively. Relative to the background period, their TSS/COD ratios increased by 89.5%, 77.2%, and 10.17%, respectively. Their peak ISS/COD ratios were 1.98, 1.66, and 0.95 (i.e., increased by 112.9%, 95.3%, and 66.67%, respectively, compared with the background period). Since the increase of the ISS/COD ratio was much larger than that of the TSS/COD ratio, we deduced that a substantial amount of the suspended solids brought in by the surface runoff was mainly inorganic solids.

### 3.3. MLVSS/MLSS Variation of Mixed Liquor

**Figure 6:**
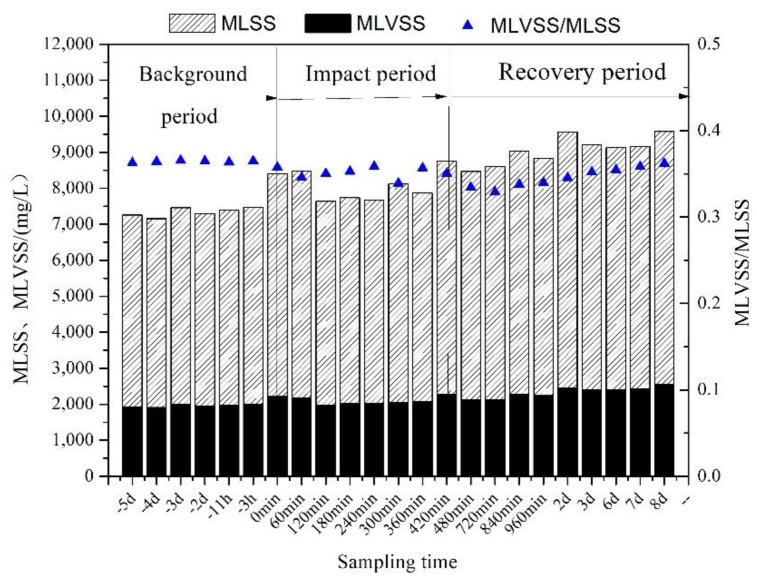
The variation of mixed liquor MLVSS/MLSS during rainfall.

After the rainfall, due to the influx of fine sediment, the sludge concentration (MLSS) increased by about 1200 mg/L, and the MLVSS/MLSS ratio of the mixed liquor decreased by 0.05. The results are shown in Figure 6.

**Figure 7:**
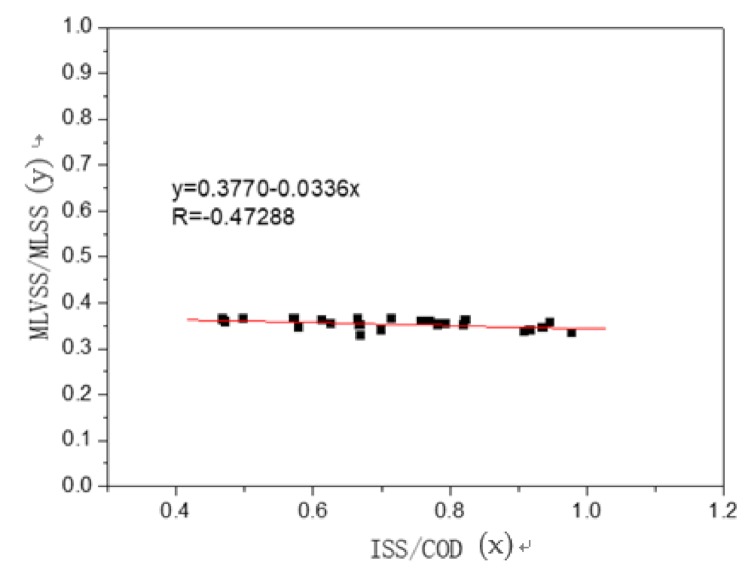
Correlation between ISS/COD and MLVSS/MLSS.

The correlation analysis between the MLVSS/MLSS ratio of the mixed liquor and ISS/COD ratio of the primary sedimentation tank effluent is shown in Figure 7. The R value was a linear correlation.

## 4. Discussions

### 4.1. The Influence of Surface Runoff on Wastewater Pollutants

The influence of surface runoff on the combined wastewater was mainly reflected in two aspects: firstly, the scouring effect caused the increase of pollutant concentration; secondly, the dilution effect of rainwater on domestic sewage. The concentrations of COD and TSS and ISS during the initial rainwater were higher while the concentrations of TP and TN were relatively low [22]. Therefore, when the surface runoff entered WWTP, the concentrations of COD, TSS, and ISS first reflected the scouring effect of initial rainwater and then reflected the dilution effect of surface runoff [23]. The effects on TN and TP were primarily dilution [24]. After the rainfall started, the average volume diameter of the influent from the grit chamber increased from 54.4 μm–71.9 μm, with the maximum value appearing at 80 min. This indicates that the concentration and particle size of suspended solids washed into the WWTP peaked at the same time when the surface runoff peaked.

### 4.2. The Differences of Removal Efficiency between the Grit Chamber and Primary Sedimentation Tank

Although both the grit chamber and primary sedimentation tank are responsible for removing suspended solids within the WWTP system, the removal rate of the primary sedimentation tank was obviously better. However, most of the suspended solids removed by the primary sedimentation tank were organic matter, which reduced the carbon source required for the subsequent biochemical treatment [25,26]. For this reason, the primary sedimentation tank was gradually removed in the SBR and oxidation ditch process, widely used in small and medium-sized cities. This resulted in a large number of inorganic solids entering the biochemical tanks, followed by their accumulation in the mixtures and at the bottom of tanks when surface runoff shocked [27,28]. The TP removal rate increased during the whole runoff impact process, which may be caused by huge amount of iron and aluminum ions transported by the surface runoff [22].

The mean TSS/COD ratio of the influent of the primary sedimentation tank was normally about 1.24, but it reached as high as 2.35 during the impact period. Compared with the typical TSS/COD ratio of 1.1 in developed countries, the TSS/COD ratio of the influent of the sewage plant was relatively high [29]. After the treatment at the primary settling tank, the TSS/COD of the effluent dropped to 1.14, which was close to the normal level. Whilst the ISS/COD of the effluent was reduced from 1.24–0.67, it was way above the typical ISS/COD ratio of 0.2 in developed countries [29]. The COD/TN ratio of the effluent was reduced from over 10 to about seven, slightly below the normal ratio of eight required for phosphorus and nitrogen removal [30]. These results indicate that the primary sedimentation tank substantially reduced the carbon source, which affected the subsequent biochemical treatment.

### 4.3. The Influence of Surface Runoff on WWTP Performance

Although the impact of a single rainfall event on the sewage plant was not obvious, the accumulation of sediments after repeated rainfall events would have serious consequences. According to previous studies, increasing sludge concentration to maintain a constant volume of MLVSS is the only way for WWTP to resist the sediment shock impact in the rainy season. After repeated rainfall shocks, the highest sludge concentration of WWTP could reach 8000 mg/L, and the MLVSS/MLSS ratio could be reduced to as low as 0.24. Although this method can enhance the sewage treatment effect, the accumulation of fine sediments in sludge will inevitably reduce the mass transfer efficiency of oxygen in the aeration tank, aggravate the wear and tear of the reflux sludge pump, and ultimately affect the sewage treatment process.

The correlation coefficient between the MLVSS/MLSS ratio of the mixed liquor and ISS/COD ratio of the primary sedimentation tank effluent during rainfall was at 0.47. This further illustrates that the concentration of influent sediment in WWTP during a rainfall period has an important influence on the MLVSS/MLSS ratio of the mixed liquor. According to the above results, the sludge concentration increased to 1200 mg/L and the MLVSS/MLSS ratio decreased to 0.05 in this single rainfall event. Moreover, this is the outcome of setting up the primary settling tank. If the primary sedimentation tank were removed, inorganic solids such as sediment during rainfall would have a profound impact on the activated sludge treatment system.

With the absence of the primary settling tank in the SBR and oxidation ditch process, there were increasing inorganic suspended solids with particle sizes of less than 200 μm (which cannot be removed by the grit chambers) flowing into the biochemical tanks. As a result, the MLVSS/MLSS ratio of the activated sludge declined significantly. For example, the MLVSS/MLSS ratio of some WWTPs hit 0.3–0.5 in China [31], which is much lower than the typical value of 0.7 [29,32].

## 5. Conclusions

(1) The influence of surface runoff on the combined wastewater is mainly reflected in two aspects: firstly, the scouring effect leads to the increase of pollutant concentration. Relative to the background period, the COD, TSS, and ISS concentrations during rainfall were higher by 30.7%, 113.5%, and 139.8%. Secondly, the dilution effect of rainwater on the concentrations of TN and TP of the domestic sewage was obvious.

(2) The surface runoff has a long-lasting effect on the grit removal capacity of the grit chamber. In the background period, the removal rates of COD, TSS, and ISS in the grit chamber were 5.21%, 12.65%, and 13.33%, respectively. In the impact period, they decreased to 0.00%, 0.00%, and 1.18%, respectively. The primary sedimentation tank demonstrated a strong impact resistance during the rainfall impact period. In the impact period, the removal rates of COD, TSS, and ISS in the primary settling tank escalated from 45.64%, 49.59%, and 63.25% to 51.52%, 73.12%, and 73.27%, respectively. Combined with the increase of the COD removal rate, it can be inferred that the primary settling tank aggravates the removal of organic carbon in sewage during rainfall, which has a negative impact on subsequent biological treatment.

(3) After the rainfall, the influx of inorganic solids (such as fine sediment) pushed the sludge concentration (MLSS) by about 1200 mg/L. The MLVSS/MLSS ratio of the mixed liquor decreased by 0.05. The correlation coefficient between the MLVSS/MLSS ratio of the mixed liquor and the ISS/COD ratio of the primary sedimentation tank effluent during rainfall was 0.47. This further confirms that the concentration of influent inorganic solids in WWTP has a significant influence on the MLVSS/MLSS ratio of the mixed liquor.

## Figures and Tables

**Table 1:** Variation range and mean value of pollutant concentration.

Treatment Facilities	Period	COD (mg/L)		TSS (mg/L)		ISS (mg/L)		TN (mg/L)		TP (mg/L)		Particle Size (μm)	
		Variation Range	Mean Value	Variation Range	Mean Value	Variation Range	Mean Value	Variation Range	Mean Value	Variation Range	Mean Value	Variation Range	Mean Value
Grit Chamber Influent	background period	512–600	557	612–735	688	440–654	518	46–59	54.9	6.31–9.67	8.28	52.09–56.43	54.41
	impact period	412–728	596	428–1469	966	409–1242	761	30–56	43.8	3.68–8.18	6.11	65.85–74.42	71.95
	recovery period	312–612	507	374–788	659	336–665	536	30–61	43.9	3.98–9.72	7.10	55.93–65.27	61.70
Grit Chamber Effluent	background period	501–596	528	534–697	601	402–554	449	44–59	51.7	7.56–10.65	8.29	51.29–55.85	54.09
	impact period	514–806	627	497–1512	971	459–1244	752	31–55	41.4	3.12–7.57	5.12	62.54–72.76	68.16
	recovery period	273–596	500	433–752	645	364–647	558	29–62	41.8	3.63–10.63	7.05	56.85–64.68	60.85
Primary Settling Tank Effluent	background period	198–355	287	236–387	303	132–205	165	33–50	41.1	4.54–7.95	5.62	52.34–55.45	55.20
	impact period	208–332	304	223–321	261	158–254	201	29–54	41.9	2.82–7.95	5.08	53.62–64.22	60.35
	recovery period	158–331	242	186–322	273	106–259	210	28–45	34.1	3.04–5.17	4.25	53.45–60.51	57.50

Note: COD: chemical oxygen demand; TSS: total suspended solids; ISS: inorganic suspended solids; TN: total nitrogen; TP: total phosphorus.

**Table 2:** The SS/COD, ISS/COD, COD/TN, and COD/TP ratios of the influent of the grit chamber.

Period	SS/COD		ISS/COD		COD/TN		COD/TP	
	Variation Range	Mean Value	Variation Range	Mean Value	Variation Range	Mean Value	Variation Range	Mean Value
background period	1.10–1.38	1.24	0.74–1.13	0.93	8.80–12.7	10.24	60.51–93.80	68.71
impact period	0.98–2.35	1.58	0.79–1.98	1.26	9.24–21.24	13.74	66.06–147.92	99.56
recovery period	1.05–1.57	1.31	0.26–1.24	1.06	9.74–15.67	11.72	52.08–106.32	75.74

**Table 3:** SS/COD, ISS/COD, COD/TN, and COD/TP of grit chamber effluent.

Period	SS/COD		ISS/COD		COD/TN		COD/TP	
	Variation Range	Mean Value	Variation Range	Mean Value	Variation Range	Mean Value	Variation Range	Mean Value
background period	1.01–1.25	1.14	0.76–1.06	0.85	8.86–11.66	10.30	52.00–62.44	59.48
impact period	0.95–2.02	1.54	0.75–1.66	1.20	10.09–22.70	15.63	82.38–225.78	133.04
recovery period	1.1–1.59	1.31	0.94–1.33	1.14	8.71–12.65	11.93	55.41–97.32	74.23

**Table 4:** SS/COD, ISS/COD, COD/TN, and COD/TP of primary settling tank effluent or AAO influent.

Period	SS/COD		ISS/COD		COD/TN		COD/TP	
	Variation Range	Mean Value	Variation Range	Mean Value	Variation Range	Mean Value	Variation Range	Mean Value
background period	0.66–1.34	1.09	0.47–0.71	0.59	3.99–10.26	7.31	39.52–61.25	51.33
impact period	0.68–1.18	0.87	0.47–0.95	0.67	5.63–9.66	7.40	38.15–104.40	59.68
recovery period	0.94–1.21	1.15	0.67–1.03	0.88	4.97–8.12	7.01	44.21–84.38	57.35
